# Histone deacetylase inhibitor thailandepsin-A activates Notch signaling and suppresses neuroendocrine cancer cell growth *in vivo*

**DOI:** 10.18632/oncotarget.19993

**Published:** 2017-08-07

**Authors:** Samuel Jang, Andrew Janssen, Zviadi Aburjania, Matthew B. Robers, April Harrison, Ajitha Dammalapati, Yi-Qiang Cheng, Herbert Chen, Renata Jaskula-Sztul

**Affiliations:** ^1^ Howard Hughes Medical Institute, Birmingham, AL, USA; ^2^ Department of Surgery, School of Medicine, University of Alabama, Birmingham, AL, USA; ^3^ Promega Corporation, Fitchburg, WI, USA; ^4^ UNT System College of Pharmacy, Department of Pharmaceutical Sciences, University of North Texas Health Science Center, Fort Worth, TX, USA

**Keywords:** thailandepsin A, histone deacetylase inhibitor, neuroendocrine cancer, carcinoid, medullary thyroid cancer

## Abstract

Novel therapies for neuroendocrine (NE) cancers are desperately needed as they frequently present as metastatic disease and cause debilitating symptoms by secreting excessive hormones. Induction of Notch isoforms has a tumor suppressive effect in NE cancer cell lines, and we have observed that histone deacetylase inhibitors (HDACi) potently activate Notch. In this study, we describe the potential for *Burkholderia thailandensis*-derived class I HDACi thailandepsin A (TDP-A) as a Notch activator and therapeutic agent against NE cancer. IC_50_ for TDP-A was determined to be 4-6 nM in NE cancer cell lines (BON, MZ-CRC-1, and TT) without cytotoxicity to lung fibroblasts. The binding characteristics of TDP-A to its target HDAC1 was examined using bioluminescence resonance energy transfer (BRET). Western blot and flow cytometry analysis showed that TDP-A induces cell cycle arrest and apoptosis in a dose-dependent manner. TDP-A dose-dependently activated the Notch pathway as measured by increasing functional CBF1-luciferase reporter signal and mRNA and protein expressions of Notch isoforms, which were attenuated by pretreatment with γ-secretase inhibitor DAPT. Furthermore, TDP-A lead to changes in expression level of downstream targets of Notch pathway and reduced expression of NE cancer markers. An *in vivo* study demonstrated that TDP-A suppressed NE cancer progression. These results show that TDP-A, as a Notch activator, is a promising agent against NE cancers.

## INTRODUCTION

Neuroendocrine (NE) cancers are a class of malignant neoplasms derived from neural crest cells. This collection of tumors encompasses multiple subtypes found in different anatomical locations, including pulmonary, gastrointestinal tract, pancreas and thyroid [1-4]. Although they are relatively rare and slow growing, NE cancers are highly metastatic and are often clinically silent until they have already metastasized, most often to the liver [5]. Furthermore, they cause debilitating symptoms by releasing supernormal concentrations of characteristic hormones into circulation, including calcitonin, somatostatin, chromogranin A (CgA), serotonin or 5-hydroxytryptamine (5-HT), and synaptophysin (SYN) [6-8]. This over secretion may lead to heart failure, but more often causes a constitution of debilitating symptoms known as carcinoid syndrome, which includes uncontrollable diarrhea, skin rashes, flushing and severe wheezing. Therefore, there is an urgent need to develop novel therapeutic agents to suppress NE tumor growth and treat symptoms.

Genetic mutations play an important role in cancer development and progression; however, epigenetic abnormalities also affect cellular phenotype and gene expression without altering the DNA sequence. Consequently, epigenetic interventions have emerged as an attractive strategy against cancers [9], as epigenetic alterations are major drivers of NE carcinogenesis [1, 10]. To target this dysregulated epigenetic control, histone deacetylase inhibitors (HDACi) garnered interest as promising antineoplastic agents since alterations in HDAC affect gene expression and contribute to cancerous transformations [11]. Others and we have shown that HDACi treatment leads to specific chromatin rearrangement and remodeling, reactivates tumor suppressor genes, promotes apoptosis, and halts tumor progression [12, 13]. The therapeutic potential of HDAC inhibitors as anticancer agents resulted in the FDA approval of four HDAC inhibitors (SAHA, FK228, PDX101, and LBH589) to date against T-cell lymphoma and multiple myeloma [14]; however, there are currently no FDA approved HDAC inhibitors against NE cancers. Specifically, we are interested in the reactivation of the Notch pathway, which is characteristically minimally active in NE cancers. Notch reinstitution in NE cancer cells has been shown to play a major role in tumor suppression, apoptosis, and alteration of the NE cancer phenotype [15-18].

Thailandepsin A (TDP-A) is a potent class I HDAC inhibitor with a promising therapeutic potential [19]. A natural product discovered through genome mining from the Gram negative bacterium *Burkholderia thailandensis* E264, TDP-A has shown antiproliferative activity in a variety of cancer cell lines at low nanomolar concentrations [20-22]. In this study, we investigate for the first time the mechanism of TDP-A as a Notch activator and its potential as a therapeutic agent against NE cancers.

## RESULTS

### TDP-A intracellular target engagement in NE cancer cells

**Figure 1:**
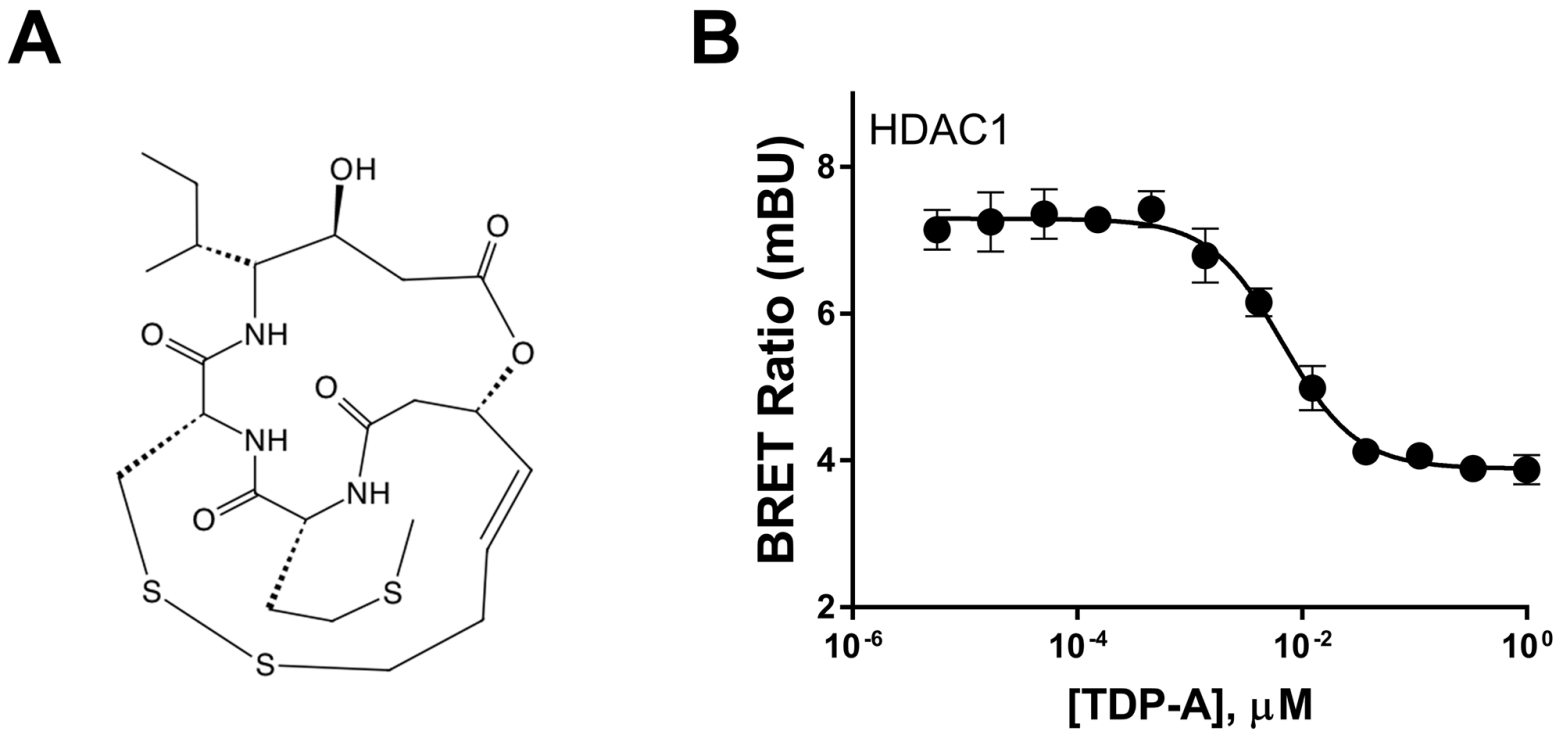
Chemical structure of thailandepsin A and its intracellular target engagement **(A)** Thailandepsin A (TDP-A), isolated from *Burkholderia thailandensis*, is a class I histone deacetylase inhibitor (HDACi). The anticancer activity is presumed to be mediated by reduction of the disulfide bond which generates a free thiol group that interacts with the catalytic site in HDACs. **(B)** Direct binding affinity of TDP-A to HDAC1 in TT cells transfected with HDAC1-NanoLuc detected by bioluminescence resonance energy transfer (BRET). Data is plotted as mean ± SD of four data points.

Biochemical assays that examine the binding kinetics of TDP-A (Figure 1A) to HDACs and its ability to inhibit them have established the natural compound as a class I HDAC inhibitor [19]. However, the live binding characteristics between TDP-A and its target HDAC1 has never been examined in a cell specific context in NE cancer cell lines. Using live cells that express a target protein fused to NanoLuc luciferase and a competitive displacement scheme, the bioluminescence resonance energy transfer (BRET) method directly examines intracellular target engagement of small molecules to its target protein [23]. In TT cells, a NE cancer cell line, TDP-A dose-dependently attenuated BRET from intracellular HDAC1-NanoLuc fusion protein (Figure 1B) in low nanomolar concentrations, where the EC_50_ was determined to be 6.8nM. This experiment demonstrates the specificity of TDP-A binding to HDAC1 within intact NE cancer cells under physiological conditions.

### TDP-A inhibits NE cancer cell proliferation *in vitro* by cell cycle arrest and apoptosis

**Figure 2:**
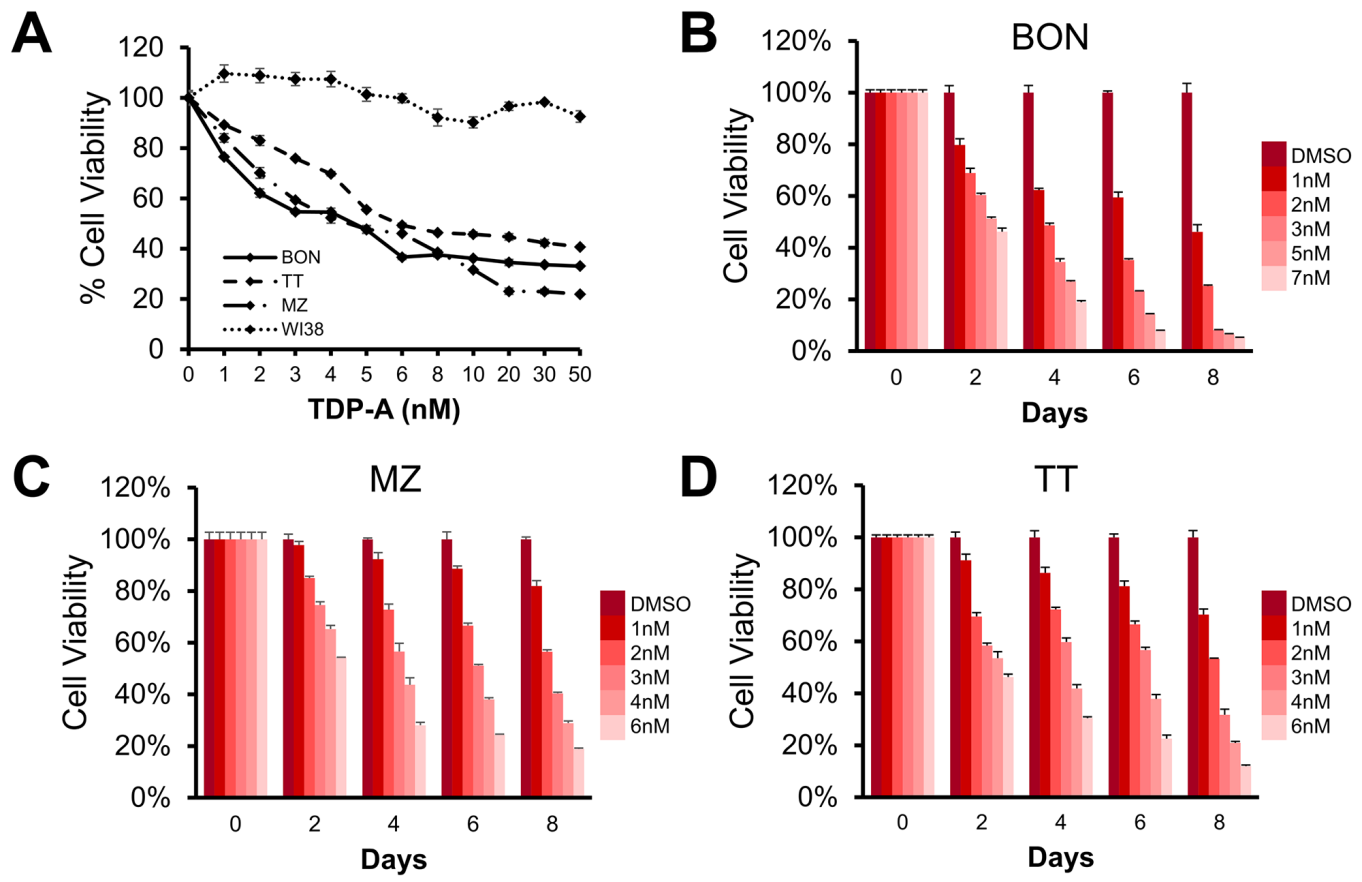
Effect of thailandepsin A (TDP-A) on neuroendocrine (NE) cancer cell viability **(A)** The IC_50_ value for TDP-A treated BON (pancreatic neuroendocrine), MZ (medullary thyroid), and TT (medullary thyroid) cancer cells was determined to be at low nanomolar concentrations (4.6, 4.5, and 6.0 nM, respectively) measured with the MTT assay after treatment for 48 hrs, while not affecting the viability of WI38 cells (lung fibroblasts). **(B-D)** TDP-A treatment reduced cell viability in a dose and time dependent manner in all three cell lines. Data is presented as mean percentage viability ± SEM normalized to DMSO vehicle control.

We then determined the effective dose of TDP-A on NE cancer cell lines BON, MZ-CRC-1, and TT. TDP-A treatment for 48 hrs exhibited a dose-dependent growth inhibition, and the IC_50_ was observed to be in 4.6, 4.5, and 6.0 nM in BON, MZ-CRC-1, and TT, respectively (Figure 2A). TDP-A had no significant effect on the viability of lung fibroblasts WI38. In a longitudinal study up to 8 days, a dose and time-dependent decrease in cell proliferation was observed (Figure 2B–2D). Statistically significant decrease was reached starting 2-day post treatment with as low as 1 nM TDP-A in all three NE cancer cell lines (P<0.01).

**Figure 3:**
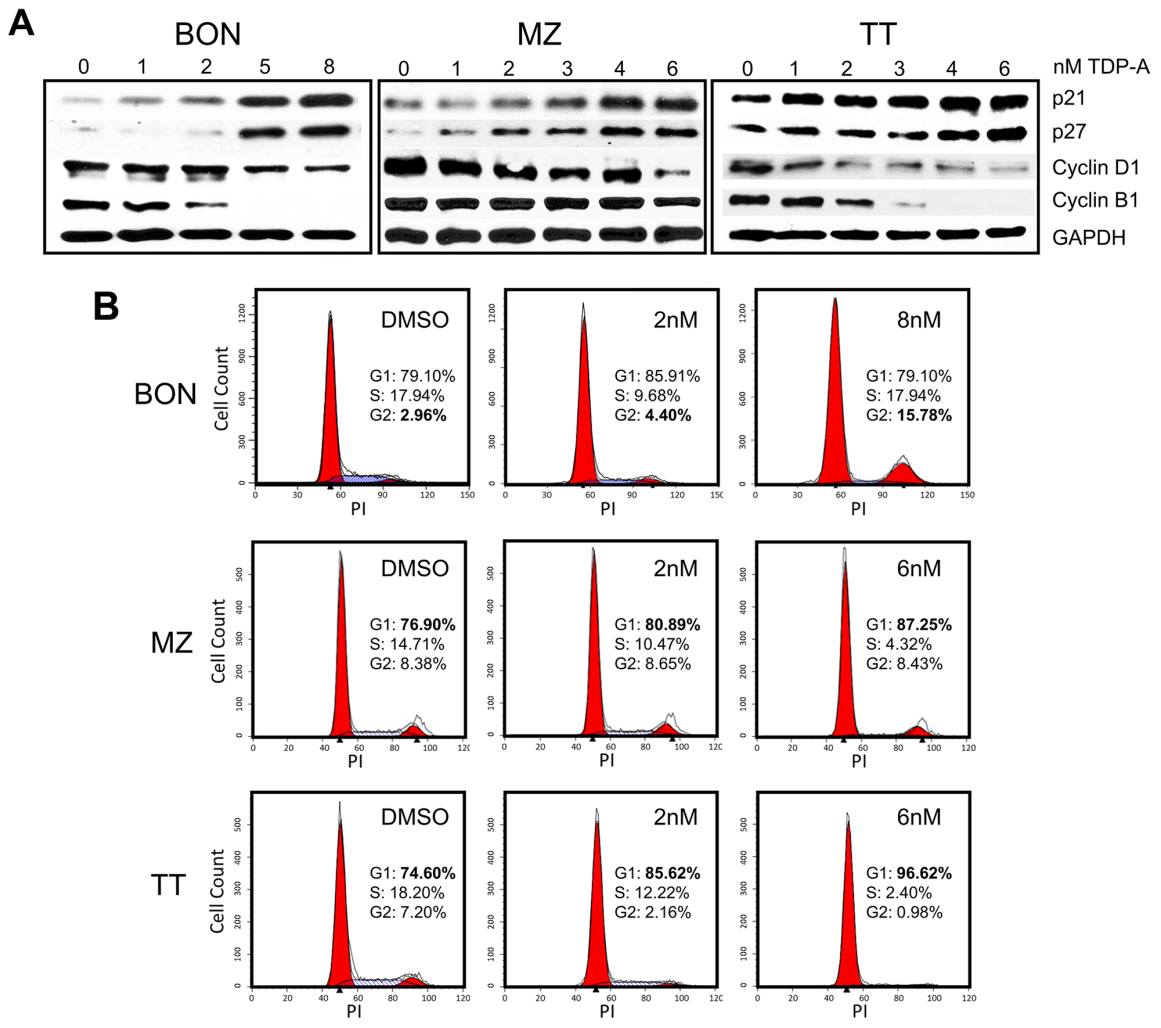
Cell cycle arrest in NE cancer cells caused by TDP-A treatment **(A)** Detection of p21, p27, cyclin B1, and cyclin D1 protein expression by Western blot in NE cancer cells treated with multiple concentrations of TDP-A (0-8nM) or vehicle control (DMSO). Equal loading was confirmed with GAPDH. **(B)** NE cancer cells were treated with two doses of TDP-A and vehicle control (DMSO) for 48 hrs, and stained with propidium iodide (3 ug/mL). Cell cycle analysis with fluorescence-activated cell sorting showed that the mechanism of growth inhibition is by cell cycle arrest at G2/M phase in BON cells and at G1 phase in MZ and TT cells.

The possible mechanism of NE cancer cell growth inhibition by TDP-A was investigated. Protein expression levels of well-established cell cycle regulators were assessed by Western blot following 48 hrs of TDP-A treatment in various concentrations (0-8uM) around the effective dose for each cell line. TDP-A treatment resulted in dose-dependent increase in cyclin-dependent kinase inhibitors p21^WAF1^ and p27^Kip1^ in all three cell lines (Figure 3A). Additionally, a decrease in expressions of cell cycle promoter cyclin B1 and D1 was observed. To further assess the effect of TDP-A on the cell cycle progression, the DNA contents in the different cell cycle phases were analyzed by propidium iodide staining after treating the NE cancer cell lines with two doses of TDP-A. Flow cytometry profiles of nuclear DNA content revealed a dose-dependent increase in number of cells in G2/M phase for BON (P<0.05) and in G1 phase for MZ-CRC-1 (P<0.05) and TT (P<0.05). All data from repeats are summarized in Figure 3B.

**Figure 4:**
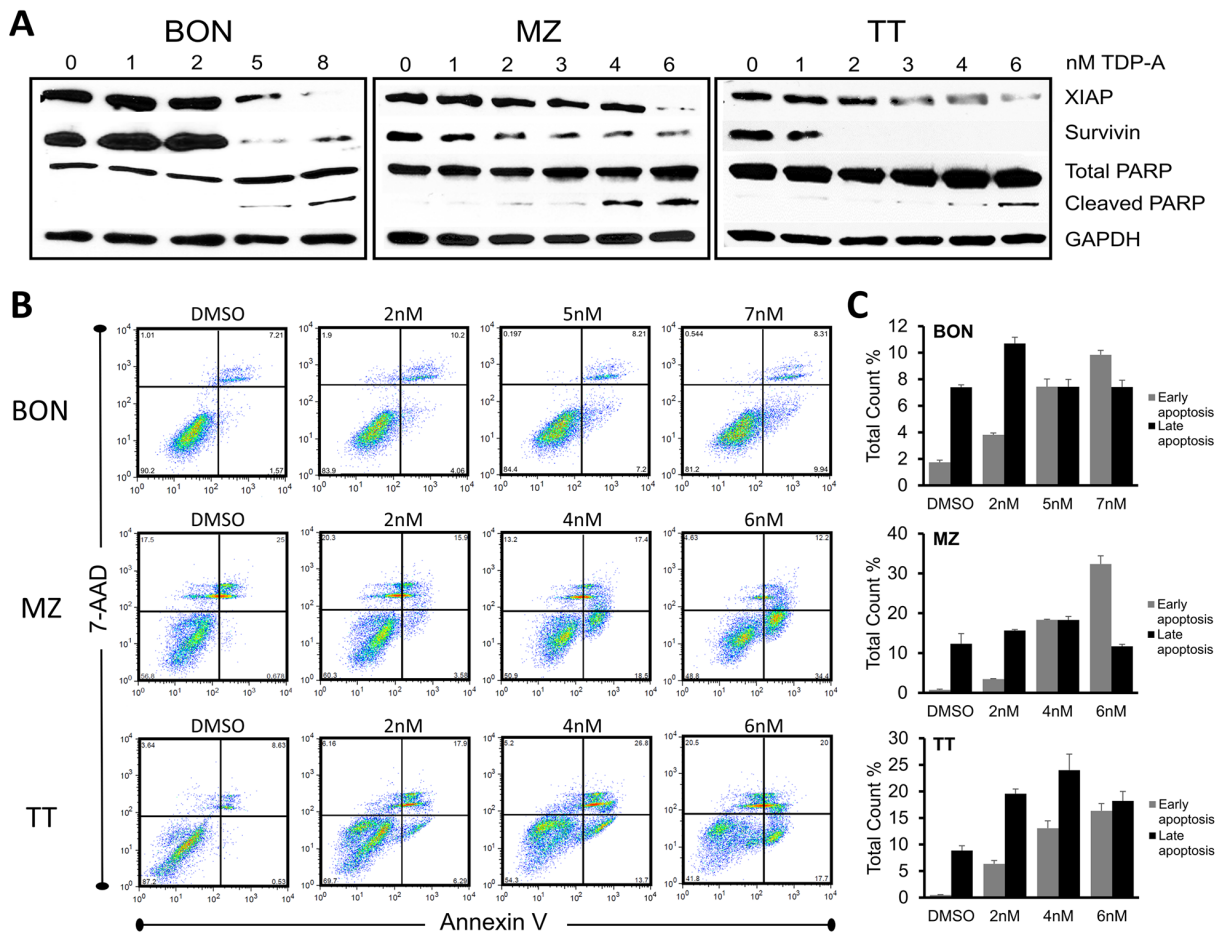
TDP-A induces apoptosis in NE cancer cells **(A)** Detection of apoptotic markers including XIAP, survivin, and total/cleaved PARP by Western blot in NE cancer cells after TDP-A treatment for 48 hrs at 5-6 concentrations around their IC_50_. DMSO vehicle control was used as negative control. GAPDH was used for loading control. **(B)** NE cancer cells were exposed to multiple concentrations of TDP-A or to vehicle control (DMSO) for 48 h before they were double stained with Annexin V and 7-AAD for flow cytometry analysis. Percentage at right upper quadrant denote the cells in late apoptotic phase and right lower quadrant denotes early apoptotic phase. The figure shows one representative experiment. Data from three repeated experiments are summarized in bar graphs **(C)**, and shown as mean percentage of total count ± SEM.

Furthermore, TDP-A caused apoptosis in the NE cancer cell lines as demonstrated by dose-dependent increase in cleavage of Poly (ADP-ribose) polymerase (PARP), a pro-apoptotic marker in the execution phase of apoptosis, and reduction in XIAP and survivin (Figure 4A). To quantify the number of cells undergoing apoptosis, NE cancer cells were double stained with Annexin V and 7-AAD after 48 hrs treatment with different concentrations (0-8uM) of TDP-A. Annexin V positivity with 7-AAD negativity represented cells in early apoptosis when analyzed by flow cytometry. This experiment provided additional evidence that TDP-A induce apoptosis in dose-dependent manner (Figure 4B shows the most representative results from repeats, and Figure 3C provides the summary). While there is a clear increase in the proportion of the early apoptotic cell population (P<0.01), the effect is not clear in the late apoptotic cell population perhaps from the cells’ full transition to necrosis. Taken together, the above results strongly suggest that TDP-A suppresses NE cancer cell growth by both cell cycle arresting at different phases depending on the cell line and by induction of apoptosis.

### TDP-A decrease NE cancer markers

**Figure 5:**
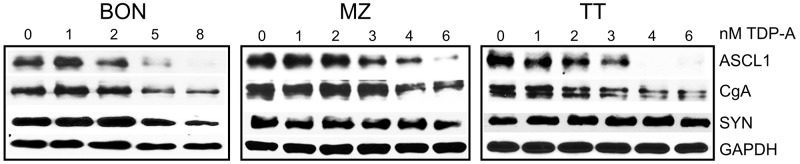
TDP-A treatment alters NE phenotypic markers Two-day TDP-A treatment results in a dose-dependent reduction of NE tumor markers achaete-scute complex like 1 (ASCL1), chromogranin A (CgA) and synaptophysin (SYN) in all three NE cancer cell lines. GAPDH was used as loading control.

Levels of NE cancer markers, which include aschaete-scute homolog 1 (ASCL1), synaptophysin (SYN), and chromogranin A (CgA), correlates with tumor burden. Their decreasing levels predicts response to treatment in patients [7, 24]. Western blot analysis revealed that treatment of NE cancer cells with TDP-A changed the NE phenotype in all three cell lines and led to dose-dependent decrease in the expression of ASCL1, SYN and CgA (Figure 5). The largest decrease was seen when cells were treated with 4 nM TDP-A. TDP-A’s capability to reduce these markers may relieve symptoms of endocrinopathy associated with NE cancers.

### TDP-A activates Notch signaling in NE cancer cells

**Figure 6:**
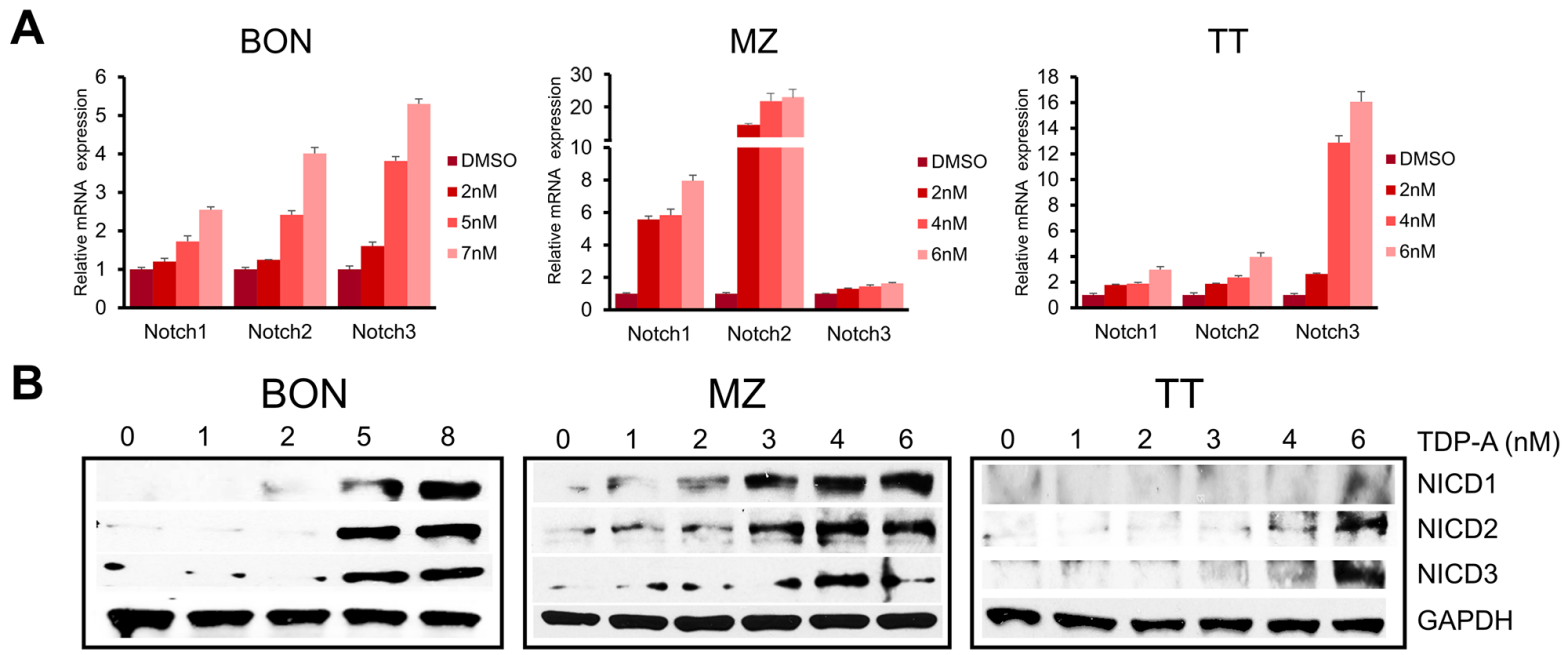
TDP-A induces Notch isoforms in NE cancer cells **(A)** TDP-A treatment resulted in significant dose dependent increase in Notch isoforms (Notch1, 2 and 3) at the mRNA level in NE cancer cells assessed by real-time RT-PCR. Data were plotted relative to cell treated with vehicle control (DMSO). All values are expressed as mean ± SEM. **(B)** Notch intracellular domain (NICD) 1-3 were detected by Western blot treated with different concentrations of TDP-A or vehicle control (DMSO). GAPDH was used as loading control.

Notch signaling is minimally active in NE cancer cells, and the induction of Notch isoforms alters the malignant NE cancer phenotype, suppresses cancer cell proliferation, and leads to apoptosis. Also, the clinical efficacy of NE cancer therapy has been shown to be correlated with Notch activation [25]. To investigate the ability of TDP-A to induce Notch isoforms, the expression of Notch1-3 was evaluated with qRT-PCR and Western blot after treating the NE cancer cell lines with various doses of TDP-A for 48 hrs. TDP-A dose dependently increased the mRNA expression of Notch1-3 in all three cell lines (Figure 6A), reaching statistical significance (P<0.05) starting at 2 nM concentration (only exception was Notch1 in BON) compared to control. Western blot analysis for the Notch1-3 intracellular domains (NICD1-3), which are created by the cleavage of the Notch receptors by γ-secretase, showed a dose-dependent increase in the three Notch isoforms in all tested cell lines after TDP-A treatment (Figure 6B).

**Figure 7:**
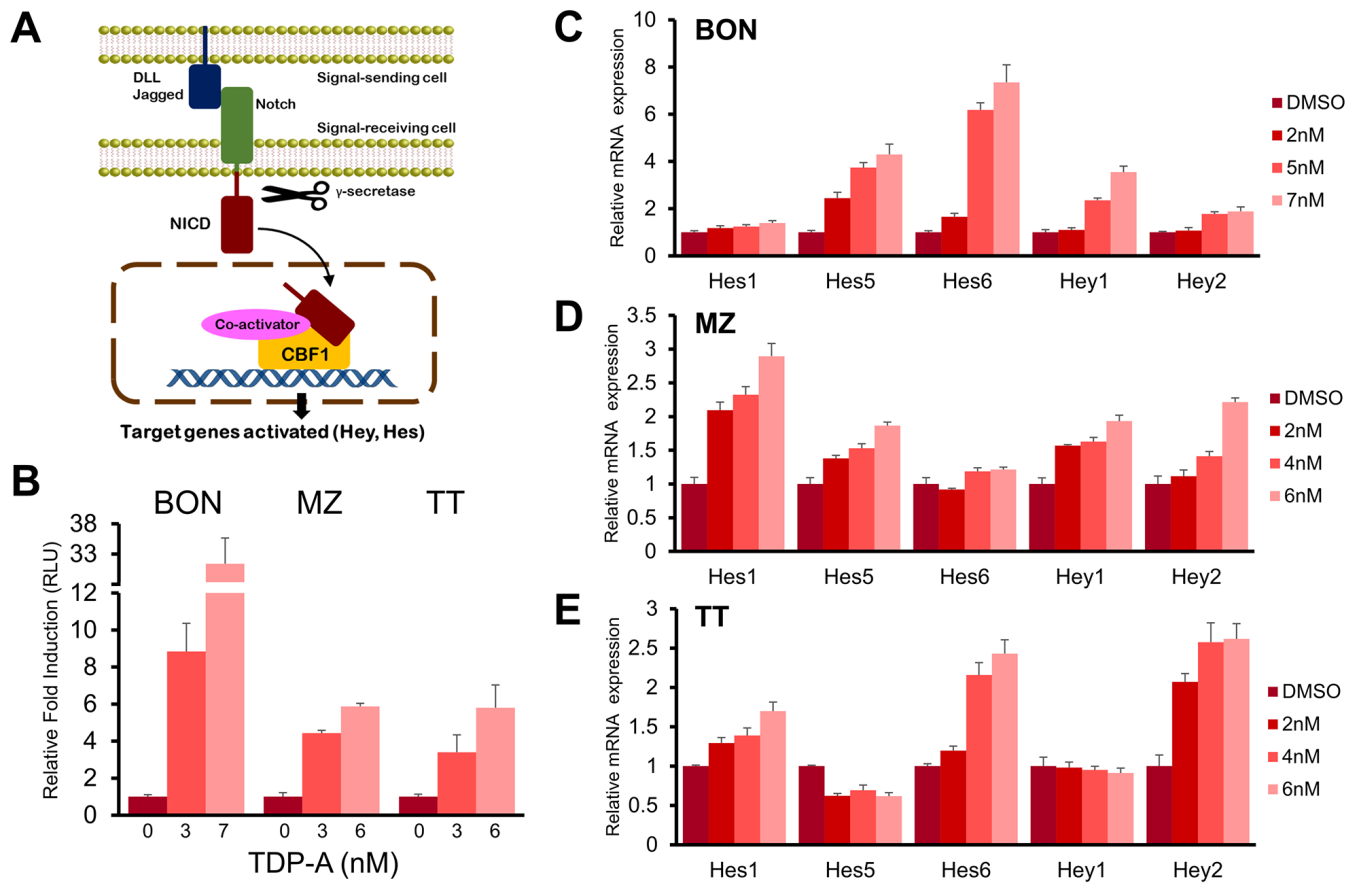
TDP-A activated Notch signaling **(A)** Canonical Notch signaling pathway that shows cleavage of Notch intracellular domain (NICD) by γ-secretase, nuclear translocation of NICD, and transcription of Notch target genes by direct interaction with CBF1. **(B)** TDP-A treatment resulted in significant increase of luciferase activity in NE cancer cells transfected with luciferase reporter plasmid containing CBF1 binding site. Luciferase activity was first normalized to β-galactosidase unit, and data were expressed relative to control cells without TDP-A treatment. **(C-E)** RT-PCR demonstrated dose dependent activation of Notch downstream targets (Hes1, Hes5, Hes6, Hey1 and Hey2 genes) in BON (C), MZ (D), and TT (E) cells at the mRNA level. Data were plotted relative to cell treated with vehicle control (DMSO). All values are expressed as mean ± SEM.

In the canonical Notch pathway, NICD translocates to the nucleus after cleavage by γ-secretase and directly binds CBF1 among other co-activators to induce the transcription of HES and HEY family of genes (Figure 7A). In order to investigate whether the Notch pathway is functionally activated in NE cancer cells by TDP-A treatment, a luciferase reporter assay that incorporates four CBF1 binding sites was used. TDP-A treatment lead to a dose-dependent induction of luciferase activity in all three NE cancer cell lines (P<0.01, Figure 7B). The greatest induction was seen in BON cells where treatment with 7 nM of TDP-A resulted in a 31-fold induction. In addition, the activation of downstream targets of Notch (Hes1, Hes5, Hes6, Hey1, and Hey2) was examined with qRT-PCR. A dose-dependent increase in the mRNA levels of most examined downstream targets was apparent in all three NE cancer cell lines (Figure 7C–7E). These results suggest that TDP-A functionally activates Notch signaling in NE cancer cells.

**Figure 8:**
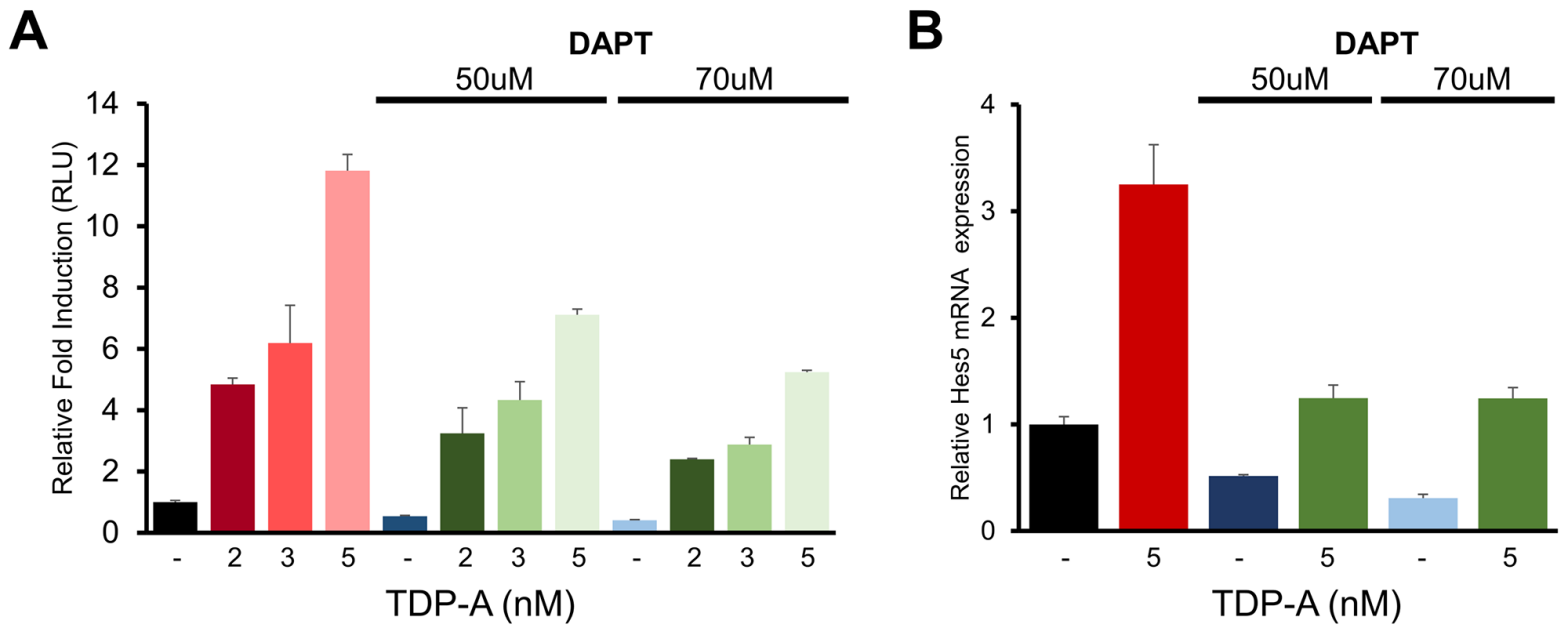
DAPT alters the effect of TDP-A on Notch activation in NE cancer cells **(A)** BON cells were first exposed to γ-secretase inhibitor DAPT and then treated with TDP-A at different concentrations or with vehicle control (DMSO). DAPT significantly blocked the effect of TDP-A on Notch-CBF1 binding in a dose dependent manner, indicating that the increase in CBF1 luciferase activity was a result of Notch induction. **(B)** DAPT also altered the TDP-A induced Hes5 mRNA expression, downstream target of Notch pathway, as measured by qRT-PCR. Data were plotted relative to cell treated with vehicle control (DMSO). All values are expressed as mean ± SEM.

To further characterize the effect of TDP-A on Notch activation seen in previous experiments, BON cells were pretreated with two different doses of DAPT before exposure to TDP-A. DAPT is a γ-secretase inhibitor that has been widely used as a specific inhibitor of Notch receptor cleavage in previous cancer research [26, 27]. DAPT effectively silenced Notch activity as examined by the CBF1-luciferase reporter assay despite TDP-A treatment (Figure 8A). While TDP-A dose-dependently induced Notch activity in DAPT pretreated cells perhaps due to residual Notch activity, the abrogating effect of DAPT was dose-dependent. In a similar manner, suppression of TDP-A mediated Notch induction by DAPT resulted in significantly reduced Hes5 mRNA expression (Figure 8B) – a Notch target gene that was induced with TDP-A treatment in BON cells. These experiments suggest that TDP-A activates the Notch pathway first by increasing the transcription of Notch receptors, followed by cleaving of the receptors by γ-secretase and forming NICD which activates CBF1, and finally leading to the activation of its downstream target genes.

### TDP-A reduces NE cancer cell progression *in vivo*

**Figure 9:**
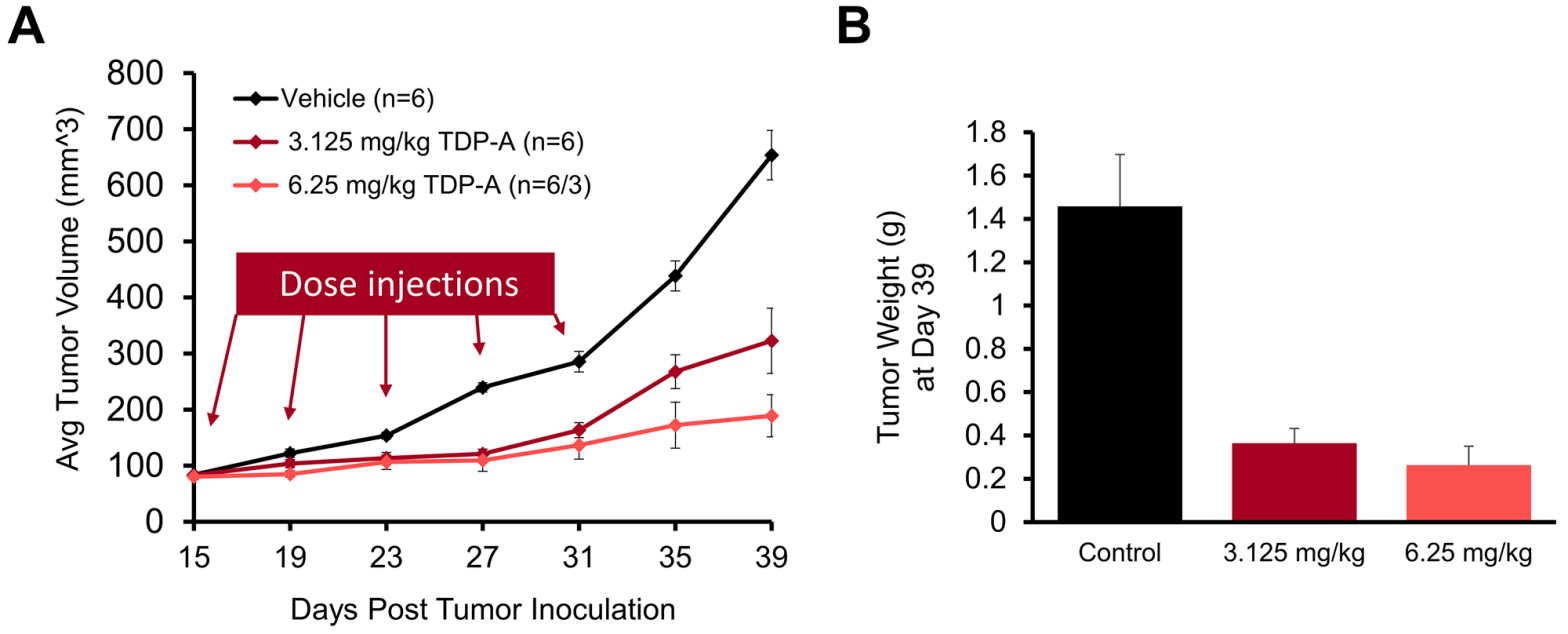
TDP-A reduces NE tumor growth *in vivo* The maximum tolerated dose of TDP-A was determined to be 6.25 mg/kg body weight. **(A)** The *in vivo* efficacy of TDP-A was tested in BON cells xenografts after subcutaneous inoculation. Intraperitoneal (IP) injections of two doses of TDP-A were performed five times every 4 days (up to day 31 post inoculation), and tumor volume was measured every 4 days. **(B)** Tumor weight was measured by resecting at the day 39 after inoculation. All values are expressed as mean ± SEM.

Finally, we evaluated the effect of TDP-A *in vivo* by using a NE cancer xenograft model. After the maximum tolerated dose was determined (6.25 mg/kg BW), nude mice bearing subcutaneous BON cell xenografts were treated with intraperitoneal injections of 3.125 mg/kg BW TDP-A (n=6), 6.25 mg/kg BW TDP-A (n=6), or vehicle control (n=6) every 4 days on days 15, 19, 23, 27, and 31 post inoculation (Figure 9A). The mean baseline tumor sizes were 83.0, 80.4, and 83.7 mm^3^ (P=0.870), respectively, and the tumor size was measured up to 39 days. There was a significant reduction in tumor volume in the TDP-A treated groups compared to the control group starting 8 days post treatment initiation (P<0.05). While the mice were undergoing treatment up to day 31, the tumors on the control mice grew 3.4 times while the lower and the higher doses of TDP-A grew 2.0 and 1.7 times, respectively. At day 39, the tumors had grown 3.9, 2.4, and 7.8 times (P=0.001) compared to their starting volumes in groups treated with lower dose of TDP-A, higher dose of TDP-A, and vehicle control, respectively. The weight of the resected tumors at day 39 post inoculation showed a significant difference between the TDP-A treated and the vehicle treated tumors (Figure 9B), with 75% reduction in the lower dose (P=0.003) and 82% reduction in the higher dose of TDP-A (P=0.012) compared to vehicle control. However, there was no significant difference between the groups treated with the two different doses of TDP-A (P=0.400).

## DISCUSSION

There is an urgency to find new therapeutic options for NE cancers since currently there is a lack of effective treatments, especially after widespread metastasis. Natural products remain the best sources of drugs and drug leads, despite the fact that many pharmaceutical companies have deemphasized natural products research in favor of high-throughput screening of combinatorial libraries during the past two decades [28-30]. Importantly, natural products have made a significant impact on FDA approved anticancer agents. A recent review highlighted that 79 of the 99 non-biologic anticancer agents are either natural products or based on a natural product scaffold [31].

We have previously observed that Notch acts as a tumor suppressor in NE cancers, where exogenous Notch expression decrease NE cancer markers and suppresses growth in carcinoid and medullary thyroid cancer cell lines [18, 32, 33]. We showed that several natural and synthetic HDAC inhibitors exerted promising anticancer activities against NE cancer cells [15, 34-38]. In the current study, we evaluated a recently discovered natural small molecule TDP-A identified as a potent inhibitor of HDACs with anti-proliferative effects on cancer cell lines [19]. We showed for the first time with the BRET method that TPD-A directly binds to HDAC1 in NE cancer cells. The same method has been used in HeLa cells to demonstrate the efficacy of the NanoBRET assay, and showed similar binding characteristics [23]. Previously performed biochemical fluorogenic assay in non-physiological condition examined the binding affinity of TDP-A to HDAC1, and showed EC_50_ of 14 nM [19]. While the value is comparable to the results of our current study (6.8 nM), the slight difference in binding affinity may stem from the cell specific environment of NE cancer cells and the sensitivity of the BRET method.

Our study shows that TDP-A has promising therapeutic effects against NE cancer cells. TDP-A inhibited NE cancer growth by both cell cycle arrest and induction of apoptosis at low nanomolar concentrations, while other HDAC inhibitors showed anticancer activities at micromolar or millimolar concentrations. These findings are consistent with previous studies evaluating the efficacy of TDP-A in other human malignancies [19-22]. In addition, we observed that TDP-A treatment of 3-6 mg/kg BW every four days effectively suppressed NE tumor growth *in vivo* with minimal side effects. Furthermore, we demonstrated that TDP-A treatment was associated with inhibition of ASCL1, CgA, and SYP, which are NE cancer biomarkers that can be used to assess clinical response to drugs [39, 40]. Our results suggest that TDP-A may not only inhibit NE cancer cell growth, but also palliate debilitating symptoms associated with endocrinopathies.

We observed a dynamic NE cancer cell death process corresponding to increasing concentrations of TDP-A treatment. G2/M phase arrest occurred in BON cells while G1 phase arrest occurred in TT and MZ-CRC-1 cells. In other studies, TDP-A induced a G2/M phase arrest in 8505C (human anaplatic thyroid cancer) cells [21] and MDA-MB-231 (human breast cancer) cells [20]. Interestingly, another natural product, thiocoraline, induces Notch in NE cancer cell, and conditional induction of NICD3 resulted in G1 phase arrest in TT cells [17, 18]. In addition, Notch signaling has been directly linked to inducing proteins that arrest the cell cycle [41]. Whether the difference in the phases of cell cycle arrest is from variations in induction of Notch isoforms, downstream effects of inhibiting HDACs, or cell specific environments remain unknown. It is clear, however, that the percentage of apoptotic cells increased with longer TDP-A treatment duration, which suggest failure of DNA repair and gradual transition to apoptosis. Increasing expression of cleaved PARP and decreases in proteins that inhibit apoptosis such as XIAP and survivin provide further evidence of TDP-A induction of apoptosis. High levels of survivin has been associated with the metastatic potential of carcinoid tumors [42], and both XIAP and survivin have been implicated in inhibiting the apoptotic pathway and conferring chemoresistance in cancer patients [43]. Taken together, the potential of TDP-A to induce cell cycle arrest, activate the apoptotic pathway, and possibly sensitize cancer cells to chemotherapy makes it an attractive agent for combinatory therapies and warrant further studies.

Similar to other HDAC inhibitors, we revealed that TDP-A induce the Notch signaling pathway in NE cancers, which has been shown to have a tumor suppressive effect. The increase in Notch1-3 mRNA expression, NICD1-3 expression, CBF1 functional activity, and the mRNA expression of Notch downstream effectors in the Hey/Hes family provide strong evidence for TDP-A’s ability to induce the tumor suppressor pathway. In a pilot phase II clinical trial, our group demonstrated that the Notch1 mRNA upregulation post valproic acid treatment, a known HDAC inhibitor, was associated with improved outcomes in patients with carcinoid tumors [25]. While we show that TDP-A target HDACs and induce the Notch pathway, TDP-A and other HDAC inhibitors may exhibit their anticancer effect by engaging other pathways. The specific downstream effects of HDAC inhibitors are areas of research interest. Additionally, the mechanism of Notch induction by TDP-A has not been elucidated.

In summary, the effects of TDP-A on NE cancers cells have not been previously characterized until now, especially as a potent activator of Notch signaling, and our results suggest that TDP-A has therapeutic potential against NE cancers.

## MATERIALS AND METHODS

### Cell culture and reagents

Three human-derived neuroendocrine (NE) cancer cell lines BON (gastrointestinal carcinoid), MZ-CRC-1 (medullary thyroid cancer), and TT (medullary thyroid cancer) were used for the current study. BON was provided by Drs. B. Mark Evers and Courtney M. Townsend Jr., (University of Texas Medical Branch, Galveston, TX), MZ-CRC-1 was provided by Dr. Gilbert Cote (MD Anderson Cancer Center, Houston, TX) and TT was provided by Dr. Barry D. Nelkin (Johns Hopkins University, Baltimore, MD). These cells lines were maintained as previously described (BON [44], MZ-CRC-1 [18], and TT [18]). WI38 (lung fibroblasts) was purchased from ATCC (Manassas, VA), and maintained in MEM supplemented with 10% FBS, 100 mg/mL penicillin and 100 mg/mL streptomycin in 5% CO_2_ at 37°C. Thailandepsin-A (TDP-A) was provided by the Cheng laboratory [19, 45], dissolved in dimethyl-sulfoxide (DMSO, Sigma-Aldrich, St. Louis, MO), and stored at -20 °C.

### Bioluminescence resonance transfer

The NanoBRET experiment was carried out in TT cells transiently transfected with plasmid DNA encoding HDAC1-Nanoluc fusion protein (Promega, Madison, WI). TT cells were transfected using Viafect according to the manufacturer’s protocol (Promega) using a 6:1 Viafect:DNA ratio. One uM NanoBRET HDAC Tracer-01 (Promega) was used to compete for the HDAC inhibitor binding site on HDAC1 against TDP-A. The TT cells were incubated with various concentrations of TDP-A for 2 hrs before NanoBRET Target Engagement substrate/inhibitor reagents (Promega) was added for BRET measurements. Refer to Robers *et al* [23] for the detailed description of reagents and methods on plasmid construction, cell transfection, competitive displacement, and BRET measurements that examine HDAC binding affinity.

### Cellular proliferation assay

BON, MZ-CRC-1, TT and WI-38 were plated in quadruplicate on 24-well plates at a density of 60,000-80,000 cells per well, incubated overnight to allow cell attachment, and treated with a range of concentrations (0–50 nM) of TDP-A for 48 hrs to determine the half-maximal inhibitory concentration (IC_50_) using 3-(4,5-dimethylthiazol-2-yl)-2,5-diphenyltetrazolium bromide (MTT) assay (Sigma-Aldrich). DMSO was used as vehicle control. On the day of measurement, the media was replaced with 250 uL of serum-free media containing 0.5 mg/mL MTT, and incubated at 37 °C for 3hrs. After adding 750 uL DMSO, the plates were measured at 540 nm using a spectrophotometer (μQuant; Bio-Tek Instruments, Winooski, VT). The IC_50_ value was determined by GraphPad Prism 6 (GraphPad Software, La Jolla, CA). In addition, proliferation curves for each NE cancer cell lines were produced in a similar manner with treatments lasting up to 8 days at doses around TDP-A’s IC_50_.

### Western blot analysis

NE cancer cells were treated with various concentrations (0-8 nM) of TDP-A for 48 hrs before protein lysates were isolated as previously described [46]. Protein concentrations were quantified by BCA Protein Assay Kit (Thermo Scientific, Waltham, MA). Denatured protein extracts were resolved by 4–15% Criterion TGX gradient gel (Bio-Rad, Hercules, CA) electrophoresis, transferred onto nitrocellulose membranes (Bio-Rad), blocked in milk (1xPBS, 5% dry skim milk, and 0.05% Tween-20) for 2 hrs, and incubated with appropriate primary antibodies overnight at 4 °C. The next day, membranes were washed and then incubated for 1 h at room temperature with horseradish peroxidase-conjugated secondary antibodies (Cell Signaling). The primary and secondary antibodies and their dilutions are provided in Supplementary Table 1. Immunoreactive protein bands were detected by Immunstar (Bio-Rad), SuperSignal West Pico, or SuperSignal West Femto reagents (Pierce Biotechnology, Rockford, IL). Immunoblot analyses were repeated at least three times. Expression levels of GAPDH was used as loading control.

### Quantitative real-time PCR

Total RNA was isolated from cultured cells 48 hrs after TDP-A treatment using an RNeasy Plus Mini kit (Qiagen, Valencia, VA), and RNA concentrations were determined by NanoDrop 1000 spectrophotometer (Thermo Scientific, Wilmington, DE). Complementary DNA was synthesized from 2 μg of total RNA using iScript cDNA Synthesis Kit (Bio-Rad). The quantitative real-time PCR was performed in triplicate on CFX Connect Real-Time PCR Detection System (Bio-Rad). The PCR primer sequences for the genes of interest are listed in Supplementary Table 2. Target gene expression was normalized to s27, and the comparative cycle threshold (ΔCt) method was used to calculate relative expression levels of target genes.

### Flow cytometry

NE cancer cells (2 x 10^5^) were treated with various concentrations (0-8 nM) of TDP-A for 48 hrs. To analyze cell cycle progression, the cells were processed as previously described [16]. Fluorescence-activated cell sorting analysis was performed using a FACSCalibur (BD Biosciences) at 488 nM, and results were analyzed with ModFitLT3.2 (Verity, Topsham, ME). After the same treatment, cells were lifted with nonenzymatic cell dissociation solution Cellstripper (Cellgro). Apoptosis induction was detected by PE Annexin V Apoptosis Detection KIT I (BD Pharmingen) according to manufacturer’s instructions and processed as previously described [47]. Data were analyzed using FlowJo V5.0 (TreeStar, Inc.). The assays were performed at least three times.

### Luciferase reporter assay

Notch functional activity was measured using a luciferase construct containing four CBF1-binding sites (4xCBF1-Luc). BON cell line was stably transfected with the CBF1-luciferase reporter construct and then treated with TDP-A (0-5 nM) with or without γ-secretase inhibitor DAPT (Sigma-Aldrich) pretreatment (50 and 70 μM) for 6 hrs. Luciferase activity (relative light unit) was measured by Monolight 3010 luminometer (Analytical Luminescence Laboratory, San Diego, CA), and presented as relative fold induction normalized to cells treated with DMSO control. To normalize for transfection efficiency, 0.5 μg of cytomegalovirus β-galactosidase was co-transfected as previously described [33].

### Animal study

Maximum tolerated dose (MTD) was determined by intraperitoneal (IP) injection of female 6-8 week old balb/c mice (National Cancer Institute, Rockville, MD) with various doses of TDP-A and then monitoring animal body weight changes for 8 consecutive days. The MTD of TDP-A was determined to be 6.25 mg/kg body weight. The reason for using intraperitoneal injection was that there is no stable oral preparation of the compound at this time. While intravenous injections could be done, we have found that IP injections were effective.

Four-week-old male athymic nude mice were obtained from Charles Rivers Laboratories (Wilmington, MD). They were allowed to become accustomed to the animal facility for one week to reduce stress after arrival. Mice were maintained under specific pathogen-free conditions. BON cells were subcutaneously inoculated into the left flank of mouse (5x10^6^ cells/animal) in 100 μL of Hanks Balanced Salt Solution (Mediatech, Manassas, VA). Fifteen days after inoculation, mice with palpable tumors were randomized into three groups (n=6 per group). The treatment groups consisted of vehicle control, 6.25 mg/kg body weight, and 3.125 mg/kg body weight. The vehicle was formulated with 60% poly (ethylene glycol)-block-poly (D, L-lactide) (PEG-*b*-PLA; 7.4k-*b*-2.3k) (Advanced Ploymer Materials Inc., Montreal, Canada), 30% PBS, and 10% EtOH. Intraperitoneal (IP) injections – delivered as 10 μl/g body weight of 0.6 mg/mL TDP-A – were performed five times every four days up to day 31 post inoculation. Tumor volumes were measured by external caliper every four days and then were calculated by the modified ellipsoidal formula: Tumor volume = ½ (length×width^2^). Tumors were resected at day 39 post inoculation and weighed. All experimental procedures were done in compliance with our animal care protocol.

### Statistical analysis

The data were analyzed using GraphPad Prism 6 and Excel (Microsoft, Redmond, WA). Unless specifically noted, continuous data were presented as mean ± SEM. Student’s t-test (two-sided) and one-way ANOVA were used to evaluate differences between two groups and among multiple groups, respectively. P<0.05 was considered as statistically significant.

## SUPPLEMENTARY MATERIALS TABLES


