# Supplementary material for: Histone deacetylase inhibitor thailandepsin-A activates Notch signaling and suppresses neuroendocrine cancer cell growth in vivo
Source: Oncotarget. 2017 Aug 7;8(41):70828–40. doi: 10.18632/oncotarget.19993 (PMC5642598; doi:10.18632/oncotarget.19993)
Supplement: Supplementary file 1 [file oncotarget-08-70828-s001.pdf]

Histone deacetylase inhibitor thailandepsin-A activates Notch signaling and suppresses neuroendocrine cancer cell growth *in vivo*

SUPPLEMENTARY MATERIALS

Supplementary Table 1: List of primary and secondary antibodies with their working dilutions

Protein	Primary antibody dilution	Primary antibody company	Secondary antibody dilution	Secondary antibody (Cell signaling)
**GAPDH**	1:10000	Trevigen	1:2000	Anti-rabbit
**p21**	1:2000	Cell Signaling	1:5000	Anti-mouse
**p27**	1:2000	Cell Signaling	1:6000	Anti-rabbit
**Cyclin D1**	1:1000	Cell Signaling	1:2000	Anti-rabbit
**Cyclin B1**	1:1000	Cell Signaling	1:3000	Anti-mouse
**XIAP**	1:1000	Cell Signaling	1:3000	Anti-rabbit
**Survivin**	1:1000	Cell Signaling	1:3000	Anti-mouse
**Cleaved PARP**	1:2000	Cell Signaling	1:6000	Anti-mouse
**ASCL1**	1:2000	BD Pharmingen	1:5000	Anti-mouse
**CgA**	1:1000	Invitrogen	1:4000	Anti-rabbit
**Synaptophysin**	1:1000	Santa Cruz	1:3000	Anti-mouse
**Notch1 (NICD1)**	1:1000	Santa Cruz	1:2000	Anti-rabbit
**Notch2 (NICD2)**	1:1000	Cell Signaling	1:2000	Anti-rabbit
**Notch3 (NICD3)**	1:1000	Santa Cruz	1:3000	Anti-rabbit

Supplementary Table 2: List of qRT-PCR primers

Gene	Forward primer	Reverse primer
Notch1	5’-GTCAACGCCGTAGATGACCT-3’	5’-TTGTTAGCCCCGTTCTTCAG-3’
Notch2	5’-TGTGACATAGCAGCCTCCAG-3’	5’-CAGGGGGCACTGACAGTAAT-3’
Notch3	5’-CCTAGACCTGGTGGACAAG-3’	5’-ACACAGTCGTAGCGGTTG-3’
HES1	5’-TTGGAGGCTTCCAGGTGGTA-3’	5’-GGCCCCGTTGGGAATG-3’
HES5	5’-ACCGCATCAACAGCAGCATT-3’	5’-AGGCTTTGCTGTGCTTCAGGT-3’
HES6	5’-AGCTCCTGAACCATCTGCTC-3’	5’-GACTCAGTTCAGCCTCAGGG-3’
HEY1	5’-CGAGGTGGAGAAGGAGAGTG-3’	5’-CTGGGTACCAGCCTTCTCAG-3’
HEY2	5’- GAACAATTACTCGGGGCAAA-3’	5’-TCAAAAGCAGTTGGCACAAG-3’
s27	5’-TCTTTAGCCATGCACAAACG-3’	5’-TTTCAGTGCTGCTTCCTCCT-3’
